# Duration of Heat Stress Effect on Invasiveness of* L. monocytogenes* Strains

**DOI:** 10.1155/2018/1457480

**Published:** 2018-10-09

**Authors:** Ewa Wałecka-Zacharska, Renata Gmyrek, Krzysztof Skowron, Katarzyna Kosek-Paszkowska, Jacek Bania

**Affiliations:** ^1^Department of Food Hygiene and Consumer Health Protection, Wrocław University of Environmental and Life Sciences, Wrocław, Poland; ^2^Department of Microbiology, Nicolaus Copernicus University in Toruń, Collegium Medicum of L. Rydygier in Bydgoszcz, Bydgoszcz, Poland

## Abstract

During food production and food conservation, as well as the passage through the human gastrointestinal (GI) tract,* L. monocytogenes* is exposed to many adverse conditions which may elicit a stress response. As a result the pathogen may become more resistant to other unpropitious factors and may change its virulence. It has been shown that low and high temperature, salt, low pH, and high pressure affect the invasion capacity of* L. monocytogenes*. However, there is a scarcity of data on the duration of the stress effect on bacterial biology, including invasiveness. The aim of this work was to determine the period during which* L. monocytogenes* invasiveness remains altered under optimal conditions following exposure of bacteria to mild heat shock stress. Ten* L. monocytogenes* strains were exposed to heat shock at 54°C for 20 minutes. Then both heat-treated and nontreated control bacteria were incubated under optimal growth conditions, 37°C, for up to 72 hours and the invasion capacity was tested. Additionally, the expression of virulence and stress response genes was investigated in 2 strains. We found that heat stress exposure significantly decreases the invasiveness of all tested strains. However, during incubation at 37°C the invasion capacity of heat-treated strains recovered to the level of nontreated controls. The observed effect was strain-dependent and lasted from less than 24 hours to 72 hours. The invasiveness of 6 out of the 10 nontreated strains decreased during incubation at 37°C. The expression of* inlAB* correlated with the increase of invasiveness but the decrease of invasiveness did not correlate with changes of the level of these transcripts.* Conclusions*. The effect of heat stress on* L. monocytogenes* invasiveness is strain-dependent and was transient, lasting up to 72 hours.

## 1. Introduction

*Listeria monocytogenes* is a Gram-positive rod-shaped bacterium widespread in the environment. As a pathogen,* L.monocytogenes* is capable of invading naturally nonphagocytic cells and crossing host barriers [1]. In humans,* L. monocytogenes* may cause mild infections such as gastroenteritis in immunocompetent individuals but also may contribute to serious, disseminated forms of infections, i.e., meningitis, abortion, and septicemia, mostly in immunocompromised individuals. Over 90% of human listeriosis is related to food contaminated with the bacteria [2]. The food production environment is a challenge for* L. monocytogenes* in which it has to deal with low and high temperatures, salt additives, and low pH [3]. Such a deleterious environment triggers in the pathogen a stress response. Consequently, alternative sigma factors are activated and a specific set of genes, responsible for survival under stress, is expressed [4]. One of the best-studied sigma factors is *σ*^B^. In* L. monocytogenes* this factor was shown to play a role under osmotic, oxidative, acid, and cold stress conditions [5]. Additionally, *σ*^B^ controls the expression of virulence genes, e.g*., inlAB*, affecting the invasion capacity of* L. monocytogenes* [6]. Thereby, stress conditions may influence bacterial virulence. To date it has been found that* L. monocytogenes* invasiveness may change in response to osmotic, heat and cold shock, low pH, or high pressure [7–11]. Additionally it has been shown that the exposure time and growth phase of bacteria can affect the stress response [9, 12]; however, there is no data describing how long the effect of stress lasts on* L. monocytogenes* invasiveness. The objective of this study was to investigate the duration of the heat stress effect on the invasiveness of 10* L. monocytogenes* strains.

## 2. Materials and Methods

### 2.1. Growth of* L. monocytogenes* Strains

The study was conducted on 10* L. monocytogenes* strains, representing 3 major lineages, 4 serogroups, and 2 sources of isolation (Table 1). Three human sporadic clinical isolates were obtained from patients hospitalized during 2000-2002 in Warsaw and Gdańsk and were kindly provided by Dr. J. Paciorek (National Institute of Hygiene, Warsaw, Poland). In brief, single colonies of* L. monocytogenes* were grown in 5 ml of Brain Heart Infusion (BHI) broth (Biocorp, Poland) at 37°C with shaking at 180 rpm for 8 hours. Then 5 *μ*L aliquots were transferred into 5 mL of fresh BHI and grown for 18 hours. To study the duration of the heat stress effect on* L. monocytogenes *invasiveness, one milliliter of bacterial culture was incubated at 54°C for 20 minutes. Then, heat-treated and nontreated bacteria (control) were incubated at 37°C for up to 72 hours and were used to infect the HT-29 human cell line and plated in duplicate onto BHI agar.

### 2.2. Cell Line and Culture Conditions

The human adenocarcinoma cell line HT-29 (CLS, Germany) was cultured in DMEM (Dulbecco's modified Eagle's medium; Sigma-Aldrich, Poznań, Poland) supplemented with 10 % heat-inactivated fetal calf serum (Invitrogen, Warsaw, Poland), 2 mM glutamine, 10 IU/mL penicillin, and 10 *μ*g/mL streptomycin (Sigma-Aldrich) at 37°C in 5 % CO_2_.

### 2.3. Plaque-Forming Assay (PFA)

HT-29 cells were grown to obtain 90% confluence in 6-well plates. Twenty-four hours before infection the medium was replaced by DMEM without antibiotics. HT-29 cells were infected with 3 to 7 log CFU of treated and nontreated bacteria for 2 hours; then the medium was replaced by DMEM containing 100 *μ*g/mL gentamicin (Sigma-Aldrich) and incubated for another 1.5 hours. Each well was then overlaid with DMEM containing 10 *μ*g/mL gentamicin and 1.2 % low-melting-point agarose (Prona, Gdańsk, Poland). After 2 days of culture the number of plaques was determined. Each assay was performed in duplicate and repeated at least three times. Invasiveness was expressed as a percentage of the number of plaques per number of log CFU deposited per well [9].

### 2.4. RNA Isolation

Bacterial pellets from 3-mililiter cultures were suspended in 90*μ*L of 0.1M Tris-HCl pH 7.4 containing 1mg/mL of lysozyme (Sigma-Aldrich) and incubated for 20 minutes at 37°C. One mL of TRI-reagent (Sigma-Aldrich) was added, and RNA was extracted according to the producer's protocol. RNA was treated with RNAse free DNAse I (Sigma-Aldrich) for 1 hour at 37°C to remove the DNA.

### 2.5. RT-PCR

cDNA synthesis was performed on 1 *μ*g of RNA using the Superscript III First Strand Synthesis System (Invitrogen) with random hexamer primers according to the manufacturer's instructions. No-RT controls, used thereafter to check for DNA contamination, were prepared from 1 *μ*g of RNA with the Superscript III First Strand Synthesis System in which no reverse transcriptase was added. A relative amount of* inlA, inlB, prfA, and sigB *transcripts was measured using the CFX Optical System (BioRad, Warsaw, Poland). Primers for* inlA* and* inlB* were from Werbrouck et al. [13], for* prfA *from Freitag et al. [14], and for* sigB *from Kazmierczak et al. [6]. For normalization of cDNA amount, the housekeeping gene* gap* was used [12, 15, 16]. Real time PCR was performed from 3 independent RNA preparations and PCRs were run in duplicate. PCRs were run in a total volume of 20 *μ*L containing 1*μ*L of appropriate cDNA, 450 nM primers for* gap* or 900 nM primers for* inlA*,* inlB, prfA*, and sigB, and 18 *μ*L of iQ SYBR Green Supermix (BioRad) using the protocol: initial denaturation at 95°C for 3 minutes, followed by 40 cycles of 95°C for 30s, 60°C for 30s, and 72°C for 45s. The specificity of PCR was evaluated by a melt curve analysis in a temperature range from 90°C to 50°C performed for each reaction. Residual DNA contamination was checked in each RNA sample by running no-RT controls. PCR efficiencies for each primer pair were determined on genomic* L. monocytogenes* DNA by running serial 5-fold dilutions of the template. Determined efficiencies were taken into account when calculating relative transcript levels according to Pfaffl [17].

### 2.6. Statistical Analysis

Each experiment of survival at 54°C, invasion, and relative expression was repeated at least three times. A two-way analysis of variance (ANOVA) was performed and the Bonferroni test was used with Statistica software (StatSoft, Poland) to determine whether statistical differences existing between different experimental groups. Significance was set at* p *level of <0.05.

## 3. Results

### 3.1. Survival of L. monocytogenes Strains at 54°C

Bacteria were incubated at 54°C for 20 minutes and the number of treated and nontreated bacteria was examined immediately after heat exposure and after 24-hour and 48-hour incubation at 37°C. The bacterial count was significantly reduced upon heating at 0 hours in 1* L. monocytogenes* strain only (L4). Following 24-hour incubation at 37°C a significant reduction of bacterial count was observed in 5 strains (L28, L41, L71, L2061, and L4). The highest differences in bacterial counts between treated and nontreated strains were noted after 48-hour incubation at 37°C. Here, a significant reduction of bacterial count was found in 7* L. monocytogenes* strains (L28, L41, L45, L71, L83, L1057, and L2061) (Table 2).

### 3.2. Effect of Heat Stress Exposure on L. monocytogenes Invasiveness

Ten* L. monocytogenes* strains were exposed to heat stress at 54°C for 20 minutes and incubated at 37°C for up to 72 hours. Then the invasion capacity was determined. Invasiveness of 6 out of 10 nontreated bacteria was found to decrease 2-25 times during incubation at 37°C. A significant reduction of invasiveness of nontreated bacteria was noted in 5 strains (L28, L41, L45, L71, and L2061) after 24 hours and in 1 strain (L56) after 48 hours of incubation.

**Figure 1:**
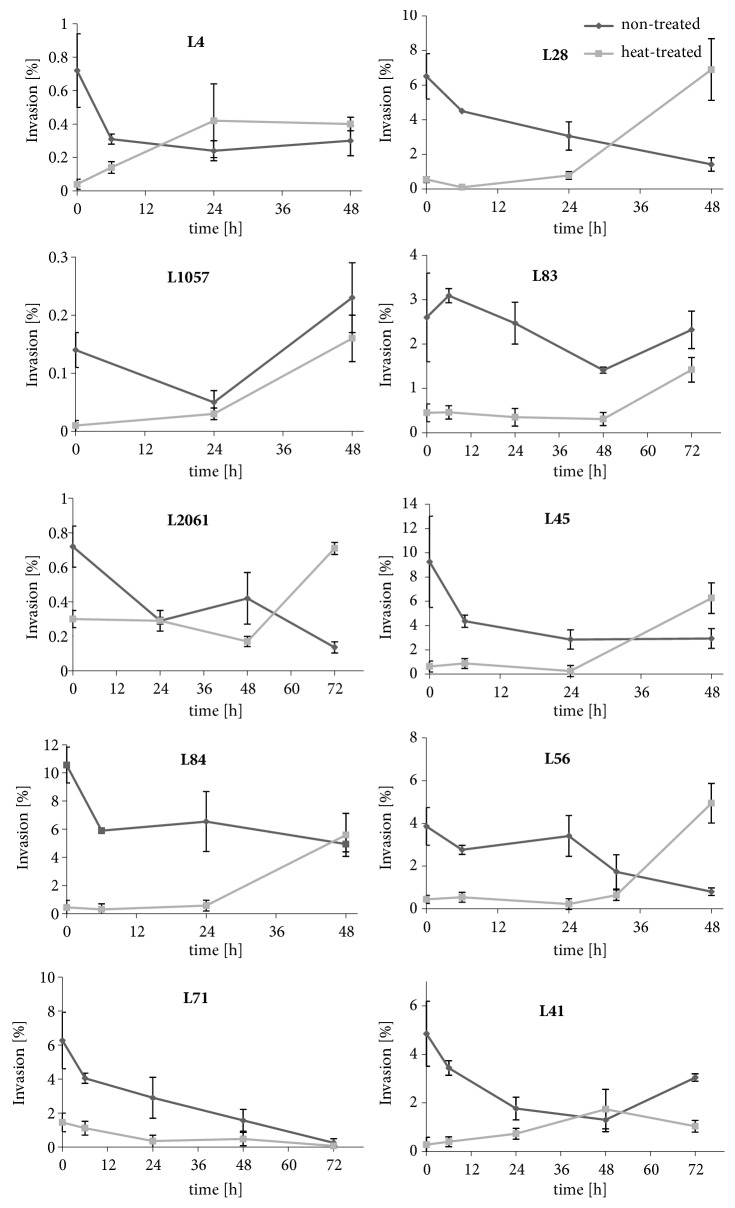
Invasiveness of 10* L. monocytogenes* strains during incubation at 37°C. The data show mean invasiveness and error bars show the standard deviations from at least three independent experiments. The data were analyzed by two-way ANOVA using the Bonferroni test.

In all strains tested a 2.4- to 25-fold reduction of invasiveness after heat exposure was observed at 0 hours (Figure 1). Invasiveness of all 10 heat-treated strains increased during incubation at 37°C reaching the value of the nontreated bacteria. The fastest increase of invasiveness after less than 24 hours was noted in the* L. monocytogenes* strain L4. Invasion capacity of two strains (*L. monocytogenes* L1057, L2061) recovered to the level of the nontreated bacteria at 24 hours of incubation at 37°C. In four strains the level of the invasiveness of the control bacteria was reached between 24 and 48 hours (L28, L41, L45, and L56) whereas for 1 strain (L84) and 2 strains (L71, L83) this level was reached after 48 and 72 hours, respectively. Moreover, 3 strains (L28, L56, and L2061) after incubation at 37°C displayed a significantly higher invasion capacity than the nontreated controls. No difference in stress effect duration on invasiveness between strains of different lineage, serotype, and origin was found.

### 3.3. Relative Expression of inlA, inlB, prfA, and sigB Genes in Response to Heat Stress

**Figure 2:**
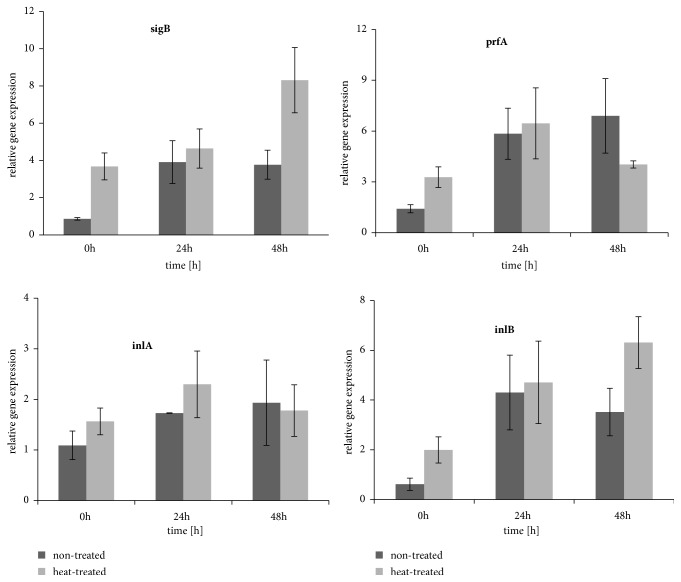
Relative change in the transcription level for* inlA*,* inlB*,* sigB*, and* prfA *genes for heat-treated and nontreated strain L71. Fold changes and SD were calculated from 3 independent RNA preparations and PCRs performed in duplicate. The data were analyzed by two-way ANOVA followed by a Bonferroni test.

The expression of selected virulence-associated genes and stress response gene, i.e.,* sigB, *was assessed on two representative* L. monocytogenes* strains L71 and L4. The invasiveness of L71 strain remained altered for the longest time after heat treatment, since it required 72 hours of incubation at 37°C, to reach an invasiveness value of that of the control strain. The invasiveness of the L4 strain reached the level of nontreated control in the shortest time, i.e., 24 hours. No significant differences in* inlA* expression upon incubation at 37°C were observed for strain L71 (Figure 2). After 24 hours an incubation increase of* inlB*,* prfA*, and* sigB *was found in the nontreated L71 strain whereas in the heat-treated strain elevated expression of* inlB* and* prfA* was noted. After 48 hours in the nontreated strain, expression of all genes did not change compared to expression after 24 hours, whereas, in the treated L71 strain, expression of* sigB* and* inlB* was significantly higher. A comparison of heat-treated and nontreated L71 strain indicated that expression of* sigB* was significantly higher following heat treatment at 0 hours and 48 hours of incubation at 37°C and expression of* inlB *was higher at 48 hours at 37°C. No correlation between expression of studied genes and invasiveness was observed for strain L71.

**Figure 3:**
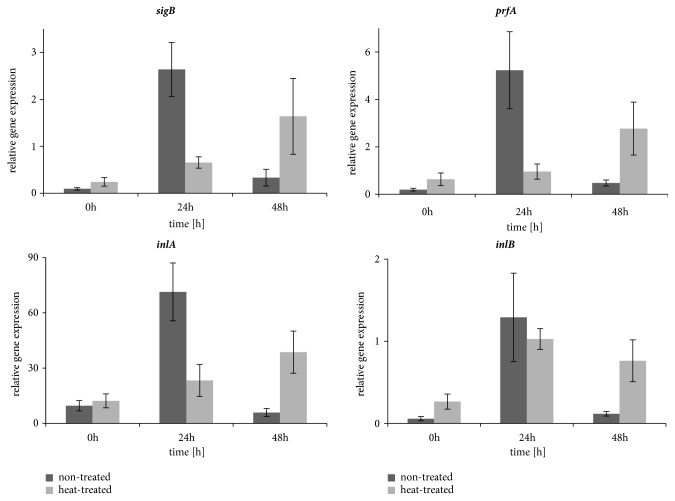
Relative change in the transcription level for* inlA*,* inlB*,* sigB*, and* prfA *genes for heat-treated and nontreated strain L4. Fold changes and SD were calculated from 3 independent RNA preparations and PCRs performed in duplicate. The data were analyzed by two-way ANOVA followed by a Bonferroni test.

In the nontreated L4 strain, expression of all tested genes was significantly higher after 24-hour incubation at 37°C and subsequently decreased after the next 24 hours of incubation (Figure 3). In the treated L4 strain an increase of* inlB* transcript was observed after 24 hours of incubation at 37°C and* prfA*,* inlA*, and* sigB* RNA increased after 48 hours. No significant differences in expression of all tested genes were found between the treated and nontreated bacteria directly after heating. After 24 hours incubation at 37°C expression of* inlA, sigB, *and* prfA *was statistically higher in the nontreated L4 strain, whereas after 48 hours expression for all tested genes was higher in the treated strain. In the heat-treated L4 strain the increase of gene expression correlated with the increase of invasiveness.

## 4. Discussion

During food processing,* L. monocytogenes* encounters many adverse conditions. In response to stress, bacteria change their metabolism which may result in resistance to other stress factors or enhanced resistance to the same kind of stress [18, 19]. Moreover, under such conditions the pathogen may modify its virulence [7, 13, 20, 21]. Little is known about stress effect duration on bacterial invasiveness. Recent work by Stollework et al. [11] has shown that the invasion capacity of bacteria exposed to HHP (high hydrostatic pressure) or heating does not change after 14-day storage at 8°C. In this study we attempted to assess the duration of heat stress effect on* L. monocytogenes* invasiveness. The mild heat treatment at 54°C was applied as previously we had found that this type of stress greatly affects* L. monocytogenes *invasiveness [9]. A temperature of 54°C has been proposed as the mean temperature of undercooked food and is encountered during the manufacture of refrigerated, processed food and during food processing in microwave ovens [22]. The study was conducted on the human adenocarcinoma cell line HT-29 since these cells were found to have a constant proliferation rate [23]. In turn, plaque forming assay applied here was shown to be the best alternative to in vivo tests to study* L. monocytogenes* virulence [24].

The heat stress effect duration on invasiveness was determined on 10* L. monocytogenes *strains. After heat treatment the bacteria were incubated at 37°C which reflects the temperature of the human GI tract.* L. monocytogenes *strains were incubated under such conditions until the invasiveness of heat-treated bacteria amounted to the value of nontreated controls. A significant reduction of bacterial counts after heating was observed in 1 strain immediately after treatment, in 5 strains after 24 hours, and in 7 strains after 48 hours. In nontreated bacteria a significant reduction during incubation at 37°C was shown in only 1 strain. It can be assumed that negative changes in bacterial cells exposed to heat shock may appear later, after 48 hours. The invasion capacity of all strains was greatly reduced in response to heat treatment. However, the invasiveness of all treated strains during incubation at 37°C reached the value of the nontreated bacteria. The effect of heat stress lasted less than 72 hours. Additionally, 3 treated strains at the end point of incubation at 37°C were more invasive than the nontreated ones. No correlation between stress effect duration on invasiveness and lineage, serotype, or strain origin was found.

60% of nontreated strains was characterized by a decrease of invasiveness during incubation at 37°C. In contrast, Bruno and Freitag [25] have shown that virulence of 1-day-old* L. monocytogenes* culture is not different from a 12-day-old culture. The discrepancy between studies can be explained by culture conditions and the test used. Additionally, a study by Bruno and Freitag was conducted in a mouse model on 1 strain only. In a few-day culture bacterial growth is limited by the availability of nutrients and oxygen. This leads to physiological changes in bacterial cells and the generation of stress response [26]. Consequently, bacteria previously exposed to stress may become more virulent in response to another unfavorable factor. This may explain differences in invasion capacity during incubation at 37°C between the heat-treated and nontreated groups.

*L. monocytogenes *in response to stress activates genes responsible for survival under adverse conditions as well as virulence genes [27–29]. Since the invasion of eukaryotic cells is determined by internalins, the expression of* inlA* and* inlB* genes was examined. Additionally, the expression of the key regulator of virulence genes* prfA* and stress response gene* sigB* was investigated. The expression of* inlAB* correlated with the increase of invasiveness. The decrease of invasiveness did not correlate with the transcript expression. To date, studies have shown that internalin expression does not always correlate with invasion capacity, suggesting the involvement of additional factors in this process [7, 12].

## 5. Conclusions

We found that the invasiveness of* L. monocytogenes* strains decreased when the bacteria were grown at 37°C. Mild heating significantly decreased the invasion capacity of* L. monocytogenes* strains at the beginning of the experiment. Further incubation of these bacteria at a temperature corresponding to those of the human body resulted in an increase of invasiveness with strain-dependent dynamics. Hence, the observed effect was transient, lasting for less than 72 hours. It can be assumed that the effect of heat treatment of 54°C for 20 minutes can alter the pathogen's invasiveness in the following 24 to 72 hours. The increase of* inlAB *transcript level correlated with the increase of invasiveness but the decrease of invasiveness did not correlate with the changes of the level of these transcripts.

## Figures and Tables

**Table 1:** * L. monocytogenes* strains used in the study.

**Strain**	**Source**	**Lineage**	**Serotype**
L28	Human	I	4b
L84	Human	I	4b
L41	Human	I	1/2b
L45	Food	I	1/2b
L56	Food	II	1/2a
L71	Food	II	1/2a
L83	Food	II	1/2a
L1057	Food	II	1/2c
L2061	Food	III	4c
L4	Food	III	4c

**Table 2:** *L. monocytogenes* strains counts (log cfu/unit) after heating at 54°C for 20 minutes and incubation at 37°C for 0 hours, 24 hours and 48 hours.

Bacterial counts reduction [log cfu/unit]			
Strain	0 hours after heating	24 hours after heating	48 hours after heating
L28	0,27±0,08	0,62±0,2 *∗*	0,66±0,02 *∗*
L84	0,31±0,06	0,46±0,12	0,71±0,23
L41	0,29±0,09	0,93±0,17*∗*	1,02±0,16 *∗*
L45	0,33±0,07	0,36±0,08	0,92±0,05*∗*
L56	0,29±0,10	0,32±0,09	0,69±0,14
L71	0,12±0,01	0,91±0,24 *∗*	0,74±0,11 *∗*
L83	0,18±0,09	0,56±0,06	1,05±0,15 *∗*
L1057	0,21±0,05	0,66±0,21	0,84±0,15 *∗*
L2061	0,65±0,14	0,99±0,22 *∗*	0,99±0,12 *∗*
L4	1,26±0,05 *∗*	0,84±0,08 *∗*	0,58±0,11

## Data Availability

The data used to support the findings of this study are available from the corresponding author upon request.
